# Prescription Analysis of Pediatric Outpatient Practice in Nagpur City

**DOI:** 10.4103/0970-0218.62564

**Published:** 2010-01

**Authors:** Anuja A Pandey, Subhash B Thakre, Prakash R Bhatkule

**Affiliations:** Department of Preventive and Social Medicine, Government Medical College, Nagpur, Maharashtra, India

**Keywords:** Error, pediatric, prescription

## Abstract

**Background:**

Medication errors are probably one of the most common types of medical errors, as medication is the most common health-care intervention. Knowing where and when errors are most likely to occur is generally felt to be the first step in trying to prevent these errors.

**Objective:**

To study prescribing patterns and errors in pediatric OPD prescriptions presenting to four community pharmacies across Nagpur city and to compare the prescription error rates across prescriber profiles.

**Materials and Methods:**

The study sample included 1376 valid pediatric OPD prescriptions presenting to four randomly selected community pharmacies in Nagpur, collected over a period of 2 months. Confirmed errors in the prescriptions were reviewed and analyzed. The core indicators for drug utilization studies, mentioned by WHO, were used to define errors.

**Results:**

The 1376 prescriptions included in the study were for a total of 3435 drugs, prescribed by 41 doctors. Fixed dose formulations dominated the prescribing pattern, many of which were irrational. Prescribing by market name was almost universal and generic prescriptions were for merely 254 (7.4%) drugs. The prescribing pattern also indicated polypharmacy with the average number of drugs per encounter of 2.5. Antibiotics were included in 1087 (79%) prescriptions, while injectable drugs were prescribed in 22 (1.6%) prescriptions. The prescription error score varied significantly across prescriber profiles.

**Conclusion:**

The findings of our study highlight the continuing crisis of the irrational drug prescribing in the country.

## Introduction

The prescribing of medicines is an integral part of the provision of health and represents a relatively safe, effective, and inexpensive mode of treatment.(1) Third-world countries spend 30-40% of their total health budget on drugs, many of which are prescribed irrationally. These countries double their expenditure on drugs every 4 years while the GNP (gross national product) doubles every 16 years.(2)

For an effective utilization of the resources spent on drugs, it is essential that the prescribing and administration of drugs be evaluated from time to time, to quantify the error in such procedures, and look for possible solutions. Medication errors are probably one of the most common types of medical errors, as medication is the most common health-care intervention. Errors can be during prescription, administration, transcription, or dispensing of the drug. In relation to prescribing, errors of omission and errors of commission have been described.(3) Errors of omission are where a prescription is incomplete in some way whereas errors of commission contain incorrect information.

Knowing where and when errors are most likely to occur is generally felt to be the first step in trying to prevent these errors. Various studies have described prescription errors in tertiary care hospitals. In contrast to hospital practice where information is easily available, the situation in community practice is different. The community pharmacist has a limited access to information related to a patient's clinical profile and laboratory tests. Hence, it is difficult for a pharmacist to use his scientific judgment in the case of incomplete or erroneous prescriptions.

Several published reports confirm that medication errors are not uncommon in pediatrics; one significant study has shown that potentially harmful medication errors may be three times more common in the pediatric population than in adults.(4) This in turn indicates that the epidemiological characteristics of medication errors may differ between adults and children. Children have therapeutic needs which probably cannot be met if medicines representing major therapeutic advances in adults are not tested and labeled for pediatric use. Once a medicine becomes available on the market for adults, it is possible to use it in children in an off-label way. Thus, the use of unlicensed and off-label medicines for children has been a common practice for decades; this does not offer children the same quality, safety, and efficacy of medicines as in adults. This situation is not consistent with the UN Convention on the Rights of the Child.(5) Hence there is a need to quantify these errors and look for possible solutions.

For the rational prescribing of medicines in pediatric age, the first model list of essential drugs for children (less than 12 years) was released in October 2007.(6) It is aimed to serve as a guideline for rational prescribing in this age group.

Considering the vital role of prescribing practices in pediatric practice, the present study was conducted to study prescribing patterns and errors in pediatric OPD prescriptions presenting to four community pharmacies across Nagpur city, and to compare the prescription error rates across prescriber profiles.

## Materials and Methods

The study was a cross-sectional survey of all prescriptions received at four pharmacies, situated in different wards of Nagpur city. Four wards of Nagpur city were randomly selected, listing of pharmacies in these areas was done, and one pharmacy in each ward was then selected by a random number table.

The study sample included pediatric outpatient prescriptions presenting to the selected four community pharmacies in Nagpur city, collected over a period of September-October 2008. The prescribing doctors were unaware that prescriptions were being audited. At the start of the study, the pharmacist of each of the selected pharmacies was informed regarding the nature of study and its utility and his or her cooperation was sought. A copy of the original prescription was used for the analysis of basic drug use and of medication errors. The information about the patient's age was gathered by the pharmacist and only those prescriptions which were meant for children below 12 years were included in the study. Prescriptions, written on scraps of paper, not containing information about the prescriber or patient, were excluded from the study. A pilot study was done to develop a mechanism to classify errors.

All the drugs prescribed were recorded including each drug dose, route, dosage form, frequency of administration, indications for which prescribed, and duration of therapy. These recorded forms were used to analyze the average number of drugs per prescription, number of encounters with antibiotics, percentage of drugs prescribed by generic name, fixed dose combinations, and whether the dosage form, frequency of administration, and duration of therapy were rational or not.

WHO specifies drug use indicators for adoption in drug utilization studies.(7) The following basic drug use indicators (core indicators) were used in the study to describe the prescribing pattern:

- Average number of drugs per encounter
- Percentage of drugs prescribed by a generic name
- Percentage of encounters with an antibiotic prescribed
- Percentage of encounters with an injection prescribed
- Percentage of drugs prescribed from the essential drug list.

The WHO model list of essential medicines for children (first list, October 2007) was used to classify drugs as essential. Errors in medication were rated on a five-point scale for each drug, where a zero score meant no error, while one point each was added for any error in drug name, dose, frequency, duration, or off-label prescribing of a drug not meant for pediatric age. These errors included errors of commission and omission. For drug dosing and frequency, values in the Monthly Index of Medical Specialties were taken as standard. For the duration of drug intake, only errors of omission were included as the duration of the intake varies with the next expected visit and with the clinical diagnosis of a patient. Prescription analysis was done by first and second authors and the five-point rating scale for prescribing error was subjected to an interrater reliability test. Kappa exceeded 0.8. The mean scores across prescriber profiles were compared. Statistical analysis of the data was done using SPSS 13.0.

## Results

The study sample consisted of 1376 valid prescriptions that met the inclusion criteria. These were for a total of 3435 drugs, the average number of drugs/prescription being 2.5 and the range being from 1 to 7. Prescriptions of 41 pediatric private practitioners could be covered under the study. No prescription from a government hospital met the inclusion criteria. Perhaps, this is due to the fact that the prescription formats of these doctors usually consist of scraps of paper with only the hospital stamp on it, and information about the prescriber was a prerequisite for inclusion in the study. Table 1 shows the number of prescriptions as per the prescriber profile. The evaluation of the prescribing indicators suggests polypharmacy, as suggested by a high average number of drugs/encounter, and in high majority, 1345 (97.7%) prescriptions, at least two or more drugs were prescribed. Fixed dose formulations dominated the prescribing pattern, many of which were irrational. Prescribing by market name was almost universal and generic prescriptions were for merely 254 (7.4%) drugs. The average number of drugs per encounter was 2.5. Antibiotics were included in 1087 (79%) prescriptions, while injectable drugs were prescribed in 22 (1.6%) prescriptions. Only 1339 out of 3435 (38.9%) drugs were included in the essential drug list. The percentage of fixed dose combinations versus single agents was 42.7%, with 1024 fixed dose combinations, which was quite high. Many of these combinations were irrational, especially those pertaining to vitamins and tonics. The prescription error score was calculated on a scale of 1-5, and mean scores of each prescriber group were compared. Error scores varied significantly across prescriber profiles, as shown in Table 2.

**Table 1:** Distribution of prescriptions as per the prescriber profile

Prescriber qualification	No. of practitioners	No. of prescriptions
MBBS with a postgraduate degree in Pediatrics (MD/DNB)	11	392
MBBS with a postgraduate diploma in Pediatrics (Diploma in Child Health)	19	817
MBBS/MBBS with other specialization (Exc. Pediatrics)	07	103
BAMS	04	64
Total	41	1376

**Table 2:** Prescription error scores across a prescriber profile

Prescriber qualification	No. of drugs prescribed	Mean error score/drug	Std. deviation
MD Pediatrics or equivalent	917	0.92	0.63
Diploma in Child Health	2053	1.17	0.82
MBBS/MBBS with other specialization (exc. ped.)	242	1.03	0.59
BAMS	223	2.11	0.91

On applying analysis of variance, it was observed that there was a significant difference in error scores across different groups of private practitioners, *P* < 0.001

## Discussion

The findings of our study highlight the continuing crisis of irrational drug prescribing in the country. Given that the vast majority of drug purchase costs are borne out of pocket, the ultimate burden of this irrational drug use falls entirely on the patient.

The number of drugs per prescription in the study area has been slightly lesser than the reported figure of 2.9 in another study conducted in Mumbai.(8) There is a lack of published studies on prescribing practices in India in this age group to conclude about this practice. However, it is preferable to keep the number of drugs per prescription as low as possible since higher figures lead to increased risk of drug interactions,(9) increased hospital cost,(10) and errors of prescribing.(11) In a similar study in Nigeria,(12) this number was 3.1 per prescription. The antibiotic use in this study has been higher than the figure of 39.6% per prescription reported by Karande *et al*.(8) The injection use was also higher than that noted in the study by Karande *et al*. in Mumbai. A total of 38.9% drugs that were prescribed conformed to a model list of essential drugs for children.

After the release of the first list of model list of essential drugs for children, in October 2007, there is no published study from India, studying the confirmation to the list. The use of essential drugs offers many advantages including cost, safety, and effectiveness. At present, however, they are not being prescribed in sufficient frequency by doctors and this needs to be improved.

Pediatrics pose a unique set of risks of medication errors,(13) predominantly because of the need to make dosage calculations, which are individually based on patients' weight, age or body surface area, and their condition. This increases the likelihood of errors, particularly dosing errors.(14) Furthermore, an incorrect recording of patients' weights and the difficulties health-care professionals have in making calculations could also contribute to incorrect dosing.(15)

Error scoring was used in this study to quantify errors in dose, frequency, duration, and off-label prescribing. The error scores were higher for practitioners who had not specialized in pediatrics. Drug doses, dosing interval, and duration of dosing were missing in high number of prescriptions. Also the information on patient's weight, which is often used for dose calculation, was missing in more than half of the prescriptions. The variation, and unsatisfactory nature of the information contained in the prescriptions are largely the result of the lack of standardization of prescription formats in the country.

Since the study was conducted in pharmacies, those prescriptions without drug indication could not be included in the study. This gives a higher estimate of the number of drugs per prescription. Along with this limitation, another limitation to this study is that over-the-counter medicines and self-medication could not be included in the study.

Although irrational prescribing is one habit which is difficult to cure, there is some evidence that interventions such as short problem-based training courses in pharmacotherapy(16) and rational use-focused workshops(17) can improve prescription behavior and skills. There is an urgent need to implement training initiatives, with support from public sources to ensure that there is no conflict of interest, to improve the prescription behavior of practitioners in India and ensure that patients receive evidence-based, cost-effective treatments for their health problems.
